# Altered Brain Microstate Dynamics in Adolescents with Narcolepsy

**DOI:** 10.3389/fnhum.2016.00369

**Published:** 2016-08-03

**Authors:** Natasha M. Drissi, Attila Szakács, Suzanne T. Witt, Anna Wretman, Martin Ulander, Henriettae Ståhlbrandt, Niklas Darin, Tove Hallböök, Anne-Marie Landtblom, Maria Engström

**Affiliations:** ^1^Department of Medical and Health Sciences (IMH), Linköping University Linköping, Sweden; ^2^Center for Medical Image Science and Visualization (CMIV), Linköping University Linköping, Sweden; ^3^Department of Paediatrics, Institute of Clinical Sciences, Sahlgrenska Academy, University of Gothenburg Gothenburg, Sweden; ^4^Department of Behavioral Science and Learning, Linköping University Linköping, Sweden; ^5^Department of Clinical and Experimental Medicine, Linköping University Linköping, Sweden; ^6^Department of Radiology, Medical Diagnostics, Highland Hospital Eksjö, Sweden; ^7^Department of Neurology, Uppsala University Uppsala, Sweden

**Keywords:** narcolepsy, default mode network, functional magnetic resonance imaging (fMRI), electroencephalography (EEG), microstates, resting state networks, orexin, sleep

## Abstract

Narcolepsy is a chronic sleep disorder caused by a loss of hypocretin-1 producing neurons in the hypothalamus. Previous neuroimaging studies have investigated brain function in narcolepsy during rest using positron emission tomography (PET) and single photon emission computed tomography (SPECT). In addition to hypothalamic and thalamic dysfunction they showed aberrant prefrontal perfusion and glucose metabolism in narcolepsy. Given these findings in brain structure and metabolism in narcolepsy, we anticipated that changes in functional magnetic resonance imaging (fMRI) resting state network (RSN) dynamics might also be apparent in patients with narcolepsy. The objective of this study was to investigate and describe brain microstate activity in adolescents with narcolepsy and correlate these to RSNs using simultaneous fMRI and electroencephalography (EEG). Sixteen adolescents (ages 13–20) with a confirmed diagnosis of narcolepsy were recruited and compared to age-matched healthy controls. Simultaneous EEG and fMRI data were collected during 10 min of wakeful rest. EEG data were analyzed for microstates, which are discrete epochs of stable global brain states obtained from topographical EEG analysis. Functional MRI data were analyzed for RSNs. Data showed that narcolepsy patients were less likely than controls to spend time in a microstate which we found to be related to the default mode network and may suggest a disruption of this network that is disease specific. We concluded that adolescents with narcolepsy have altered resting state brain dynamics.

## Introduction

Narcolepsy is a chronic sleep disorder, characterized by excessive daytime sleepiness with frequent uncontrollable sleep attacks (Silber et al., 2002; Dauvilliers et al., 2007). Other symptoms include rapid eye movement (REM) sleep abnormalities such as sleep paralysis, hypnagogic (upon falling asleep) or hypnopompic (upon awakening) hallucinations, and nocturnal dyssomnia with fragmented sleep and frequent awakenings. Most narcolepsy patients also suffer from cataplexy, a sudden reduction or loss of muscular tone not accompanied by loss of consciousness caused by the loss of hypocretin-1 (orexin) producing neurons in the lateral hypothalamus (Lin et al., 1999; Thannickal et al., 2000; Nishino et al., 2010). In addition to the sleep-related changes summarized here, the loss of hypocretin-1 is also thought to be an underlying cause to the changes in cognition observed in patients with narcolepsy (Fulda and Schulz, 2001; Rieger et al., 2003; Naumann et al., 2006; Bayard et al., 2012).

Results from previous functional and structural neuroimaging studies would suggest that the loss of hypocretin-1 has numerous downstream effects in terms of both resting state glucose metabolism and perfusion and reduction in cortical gray matter. Specifically, studies investigating narcolepsy with positron emission tomography (PET) and single photon emission computed tomography (SPECT) have observed aberrant perfusion and glucose metabolism in the hypothalamus and thalamus, as well as in prefrontal cortex (PFC; Joo et al., 2004, 2005; Hong et al., 2006; Dauvilliers et al., 2010). A very recent PET study in a large co-hort of adolescents with type 1 narcolepsy further observed that the hypo- and hypermetabolism in many of these cortico-frontal and subcortical brain regions also exhibited significant correlations with performance on a number of neurocognitive tests (Huang et al., 2016). These findings parallel those found in structural neuroimaging studies, where a reduction of cortical gray matter in frontotemporal areas has been observed (Kaufmann et al., 2002; Brenneis et al., 2005; Scherfler et al., 2012; for a more comprehensive review of neuroimaging in narcolepsy we refer readers to the previously published work of Dang-Vu et al., 2009; Dang-Vu, 2013).

In the present study we aimed to further explore resting state brain function (as opposed to sleep) in adolescents with narcolepsy by combining resting state functional magnetic resonance imaging (rsfMRI) and electroencephalography (EEG), taking advantage of both the superior neuroanatomical localization of fMRI and the millisecond-range temporal resolution of EEG. It is currently thought that the momentary, global functional state of the brain is reflected by its electrical field configuration. These electrical field configurations have been shown to change discontinuously, exhibiting periods of quasi stability on the order of 100 ms before abruptly transitioning to another configuration (Lehmann et al., 1987, 2009; Michel et al., 2009). The periods of quasi stability have been termed “microstates”, are thought to arise from coordinated activity of neural assemblies originating from large areas of the cortex, and have distinct scalp topographies (Khanna et al., 2015). Given the time scale on which EEG microstates exist, it has been proposed that they may qualify as the basic blocks of mentation or “atoms of thought and emotion” (Lehmann, 1990; Strik et al., 1995; Lehmann et al., 2004, 2005; Lehmann and Michel, 2011). In addition to changes to microstate topographies observed in several neurologic and neuropsychiatric diseases such as schizophrenia (Andreou et al., 2014), Tourette syndrome (Stevens et al., 1996), panic disorder (Kikuchi et al., 2011), and depression (Strik et al., 1995), the temporal characteristics of EEG microstates have also been used to differentiate between diseased and normal populations. In defining these temporal characteristics, Koenig et al. (2005) proposed that the *mean duration* of a microstate reflected the stability of its underlying neural assemblies. The *frequency of occurrence* of a microstate might indicate the tendency of its underlying neural generators to be active. The *ratio of total time* covered and *global explained variance (GEV)* of a given microstate are both thought to reflect the relative time coverage of its underlying neural generators compared to others. Finally, the *transition probability* from one microstate to another can be interpreted as an encoded sequential activation of the neural assemblies that generate the microstates. Previous studies in diseased populations such as schizophrenia (e.g., Lehmann et al., 2005; Andreou et al., 2014), panic disorder (Kikuchi et al., 2011), depression (Strik et al., 1995), Tourette syndrome (Stevens et al., 1996), Alzheimer’s-related dementia (e.g., Dierks et al., 1997; Stevens and Kircher, 1998), and frontotemporal dementia (Nishida et al., 2013) have all observed changes in mean duration, frequency of occurrence, ratio of total time, and transition probability in patients relative to controls, which they interpreted as underlying changes in resting state brain dynamics characteristic of the disease (see Khanna et al., 2015 for a more complete review of the current resting state EEG microstate literature).

Resting state EEG studies have typically found four consistently observable microstates, thought to originate from the abstract thoughts that typically arise during unstructured rest (Yuan et al., 2012) and whose topographies have been arbitrarily labeled A, B, C, and D (Wackermann et al., 1993; Pascual-Marqui et al., 1995; Koenig et al., 2002). To further investigate the neural origins of EEG microstates, several more recent simultaneous resting state EEG and fMRI studies have shown that the occurrence of individual microstates correlated with various fMRI resting state networks (RSNs; Britz et al., 2010; Musso et al., 2010; Yuan et al., 2012). However, given the diverse methodology employed by these studies with some using spatial correlation (e.g., Britz et al., 2010; Musso et al., 2010; Van de Ville et al., 2010) and at least one other using temporal correlation (e.g., Yuan et al., 2012), no consistent relationships between individual microstates and fMRI-derived RSNs yet exist.

Resting state fMRI also gives information about spontaneous brain activity by measuring blood oxygen level dependent (BOLD) signal fluctuations (Raichle et al., 2001; Greicius et al., 2003; Birn et al., 2008; Fox et al., 2009; He and Raichle, 2009; Shimony et al., 2009). These resting state BOLD signal fluctuations are thought to represent spontaneous and unstructured thought processes, albeit on a much slower time scale. More traditional functional connectivity analyses, such as independent component analysis (ICA) or seed-voxel analysis, of these signal fluctuations have yielded a predictable set of temporally stable RSNs (Damoiseaux et al., 2006; Mantini et al., 2007), many of which have been linked to cognitive, sensorimotor, and emotional functions (Laird et al., 2011).

To our knowledge, no study has investigated brain function in narcolepsy during unstructured resting state using simultaneous fMRI-EEG before. Given the diverse and pervasive changes in brain structure and metabolism previously reported in narcolepsy, we anticipated that changes in RSN dynamics might also be apparent in patients with narcolepsy. A recent study by Kuhn et al. (2015) investigated sleep related brain function using EEG microstate analysis in adults with narcolepsy and found only reduced mean duration of the microstates in patients compared with controls which they interpreted as evidence of instability; no differences in GEV, ratio of total time, or transition probability between patients and controls for any microstate during either wakefulness or any sleep stage were otherwise noted. Additionally, the authors (Kuhn et al., 2015) made no attempt to correlate the microstate findings to fMRI derived RSNs beyond citing previously published work (e.g., Britz et al., 2010; Van de Ville et al., 2010). The purposes of the current study were twofold: (1) to investigate and describe brain microstate dynamics in adolescents with predominantly type 1 narcolepsy; and (2) to temporally correlate these to fMRI-derived RSNs acquired in the same subjects using simultaneous fMRI-EEG. The results were compared with age-matched healthy controls. We hypothesized that we would see differences in microstate dynamics that may suggest instability in underlying resting state brain function, such as reduced mean duration of the microstates. Additionally, we sought to determine whether temporal correlations between these EEG microstates and any RSNs exist that might help us better understand the nature of many of the cognitive deficits observed in patients with narcolepsy.

## Materials and Methods

### Participants

Twenty-one participants with narcolepsy were recruited from a population-based study in western Sweden (*n* = 15; Szakács et al., 2013, 2015) and from pediatric clinics in the county of Östergötland (*n* = 6). Inclusion criteria for patients were a confirmed diagnosis of narcolepsy and being between 13–20 years of age at time of enrollment. Data from five narcolepsy patients were excluded from further analyses due to equipment malfunction (*n* = 2), participant fatigue (*n* = 1), and orthodontia (*n* = 2). This left a final sample size of 16 participants with narcolepsy (14–20 years; 11F/5M).

Narcolepsy diagnoses were based on the classification codes of the Swedish version of the International Classification of Diseases, Tenth Revision (ICD-10) and the diagnostic criteria for narcolepsy according to the 2005 International Classification of Sleep Disorders (American Academy of Sleep Medicine, 2005). Given the updated diagnostic classification system for narcolepsy released in 2014 by the AASM (American Academy of Sleep Medicine, 2014), the narcolepsy patients were retrospectively diagnosed as being either type 1 or type 2. All patients met the diagnostic criteria of type 1 narcolepsy, except one (patient 16) who did not have cataplexy and lacked measurement of CSF-hypocretin. Additionally, all patients except one fulfilled the criteria for narcolepsy as assessed using the Multiple Sleep Latency Test (MSLT; Carskadon et al., 1986). This patient did not fall asleep during the testing but had CSF-hypocretin < 110 pg/mL and was HLA-DQB1^*^0602 positive. Based on these latter two findings, the patient was not excluded from inclusion in the study. All patients were assessed for cognitive disabilities by a neuropsychologist using either the Wechsler Preschool and Primary Scale of Intelligence Revised, 3rd edition (WPPSI-III; Wechsler, 1967) or the Wechsler Intelligence Scale for Children, 3rd/4th edition (WISC-IV; Wechsler, 2003), depending on the age of the patient. No patients were excluded due to the presence of a measureable cognitive disability. The demographic, clinical, and laboratory characteristics of the 16 participants with narcolepsy are presented in Table 1.

**Table 1:** **Adolescents with narcolepsy, demographics, clinical and laboratory characteristics**.

Patient	Gender	Age at scan (y)	Age at onset (y)	Narcolepsy duration (y)	BMI	Co-morbidity	Pandemrix vaccine	HLA DQB1*0602	CSF-Hcrt (pg/ml)	MSLT	Narcolepsy subtype	Medications
1	F	16.5	11	5	20.2	0	No	Positive	81	Positive	1	Methylphenidate, Fluoxetine
3	M	14.1	10	4	24.2	Depression	Yes	Positive	<10	Positive	1	Methylphenidate
5	F	16.7	13	3	25.4	0^c^	Yes	Positive	<10	Positive	1	Methylphenidate, Modafinil, Fluoxetine
6	F	18.3	15	3	22.3	Depression	Yes	Positive	79	Positive	1	None
8	M	19	15	4	27.7	Depression	Yes	Unknown	<10	Positive	1	Methylphenidate
9	M	18.4	15	3	23.9	Depression	Yes	Positive	49	Positive	1	Methylphenidate
10	F	19.5	15	4	24.4	Depression	Yes	Unknown	Unknown	Positive	1	Methylphenidate, Sertraline
11	F	18.3	15	3	22.5	0	Yes	Positive	18	Positive	1	None
12	F	20.2	16	4	25.2	0	Yes	Positive	<10	Positive	1	Notknown
13	M	12.2	8	5	Unknown	0	Yes	Positive	<10	Unknown	1	Notknown
15	M	15.3	13	2	27.4	Depression	Yes	Positive	58	Positive	1	Methylphenidate, Fluoxetine
16	F	17.9	14	3	41.4	0	Yes	Positive	Unknown	Positive	2	Modafinil
18	F	14.3	11	3	16.2	0	Yes	Positive	Unknown	Positive	1	Modafinil, Fluoxetine
19	F	14.3	10	4	20.9	0^a^	Yes	Positive	10	Positive	1	Methylphenidate
20	F	17.3	13	5	38.4	ADHD^b^	Yes	Positive	Unknown	Positive	1	Methylphenidate, Fluoxetine
21	F	16.2	12	4	23.7	0^a^	Yes	Positive	<10	Positive	1	Methylphenidate, Fluoxetine, Zolpidem

*Abbreviations: ADHD, Attention Deficit Hyperactivity Disorder; CSF-Hcrt, Cerebrospinal Fluid Hypocretine; BMI; Body Mass Index; F, female; M, male. ^a^According to DSM-IV (American Psychiatric Association). ^b^ADHD was present before onset of narcolepsy. ^c^Not evaluated according to DSM-IV*.

Sixteen age and gender matched healthy controls (13–20 years; 10F/6M) were recruited by advertisement. Controls were confirmed to have no medical history of neuro-pathological diseases or mental illness by questionnaires and interviews prior to examination. The study was performed in accordance with the Helsinki Declaration and approved by the Regional Ethical Review Board in Linköping, Sweden, and all participants gave informed consent to participate (2013/99-31).

### Procedure

All participants were monitored with actigraphy (Sense Wear, Body Media, Inc., Pittsburgh, PA, USA) 1 week before the fMRI-EEG examination. Participants were asked to fill in a questionnaire based on the Stanford Sleep Inventory^1^ that contained questions about sleep habits and cataplexy-related symptoms. Results from the cataplexy questionnaire and the Epworth Sleepiness Scale (ESS; Johns, 1991) are reported in Table 2. Narcolepsy patients were allowed to take their prescribed medications prior to the exam. Fatigue, depression, anxiety and sleepiness were measured directly before fMRI-EEG with visual analog scales (VAS). Before entering the MRI scanner, the EEG electrodes were positioned and controlled for proper impedance threshold (<10 kΩ).

**Table 2:** **Patient data on day of examination**.

Patient	ESS score (0–24)	Cataplexy score (0–28)	Sleep efficacy (%)
1	11	8	83
3	14	4	37
5	14	11	61
6	20	16	69
8	16	18	64
9	16	12	74
10	19	22	79
11	11	16	67
12	15	20	50
13	19	12	65
15	18	15	54
16	17	1	80
18	18	11	42
19	10	5	82
20	15	23	61
21	21	0	39

*Table shows patient data on the day of the examination. ESS, Epworth Sleepiness Scale; Sleep efficacy is the proportion of sleep in the episode potentially filled by sleep*.

### MRI

All MR images were acquired on a 3T Philips Ingenia (Philips Healthcare, Best, Netherlands) located at the Center for Medical Image Science and Visualization (CMIV) at Linköping University, Sweden. Images were acquired using a 32-channel head coil with a single-shot, gradient-echo, echo-planar imaging (EPI) sequence (repetition time (TR)/echo time (TE): 2200/35 ms; flip angle: 77°; field of view (FOV): 240 × 240 mm; matrix: 80 × 80; voxel size: 3 × 3 × 3 mm^3^; no gap; SENSE factor: 2) that effectively covered the whole brain in 2.2 s. Simultaneous EEG was also acquired during the scan as described in Section “EEG Recording”. During the resting-state scan, participants were instructed to lie still with their eyes closed and given no special instructions to stay awake. The eyes-closed resting state scan consisted of 270 time points, with a total scan time of approximately 10 min.

#### Radiology Findings

T1-weighted images were acquired for all participants to ensure that they were otherwise free from any obvious pathologic abnormalities and were reviewed by a radiologist. One narcolepsy patient was found to have an arachnoid cyst in the frontal lobe, which was known prior to inclusion. No other participant was found to have any abnormality.

### EEG Recording

For EEG recording, MR-compatible caps (Easycap, Brain Products GmbH, Gilching, Germany) with 64 silver/silver chloride (Ag/AgCl) electrodes were used. A bi-polar montage was used, based on 10–20 system positions. Electrocardiography (ECG) was measured from a separate electrode placed on the left side of the participants’ backs. The EEG was recorded (Vision recorder, Brain Products) inside the MRI scanner using a 5 kHz sampling rate, 32 mV input range and 0.1–250 Hz bandpass filters (BrainVision Recorder manual; software version 1.20.0001). MR-compatible Brain Amp amplifiers were placed in the scanner bore (Brain Products GmbH, Gilching, Germany), and optical cables passed through the wall of the scanner room to the recording equipment. A Transistor-Transistor Logic (TTL) pulse sent by the MRI scanner was used to mark the start of the fMRI resting state scan on the EEG recording.

### EEG Data Preprocessing

The EEG signal recorded during the rsfMRI scan was corrected for the MRI gradient and cardioballistic artifacts using standard template subtraction procedures (Allen et al., 1998). ICA was then used to identify common artifact components such as eye movement, shoulder/neck movements, and some persisting cardioballistic artifacts. Any remaining artifacts were rejected through visual inspection (Delorme and Makeig, 2004). Data were down sampled to 125 Hz and bandpass filtered between 1–40 Hz. All EEG preprocessing steps were performed using Analyzer 2.0 from Brain Products.

### Microstate Analysis

As both the theory and practice of microstate analysis have been previously published in much greater detail elsewhere (e.g., Murray et al., 2008), the following is only meant to be a summary of the more salient features of the microstate analysis performed for the current study.

The moment-to-moment strength of the electric field across the entire brain can be represented in terms of the Global Field Power (GFP), typically defined as the standard deviation of all electrodes at a given time point. Local GFP maxima have been shown to represent instants of strongest field strength and highest topographic signal-to-noise ratio. In microstate analysis, the topographies of local GFP maxima are considered to be discrete EEG microstates (Khanna et al., 2015). After identifying of the peaks of GFP (Pascual-Marqui et al., 1995), peaks including noisy data (e.g., movement artifact unable to be removed by the EEG preprocessing steps outlined in “EEG Data Preprocessing” Section) were visually excluded, and the remaining peaks were extracted and submitted to a modified K-means clustering analysis.

In clustering analysis of EEG microstates, the topographies of all GFP maxima are simultaneously extracted and entered into a clustering algorithm. The clustering algorithm is used to group these topographies into a small set of classes based on topographic similarity, regardless of their order of appearance. The topography at each GFP maxima is then labeled as one of these classes, and the EEG signal is re-expressed as a sequence of microstate classes. To determine the optimal number of classes or EEG microstates, a cross validation criterion was used. The cross-validation criterion objectively indicates the minimum amount of topographies to explain the maximum of the variance and yielded similar topographies across all subjects (Pascual-Marqui et al., 1995). Topographic similarity was then verified using a topographical ANOVA, which is a spatial correlation analysis. We note that resting state EEG microstate topographies are polarity invariant (Lehmann et al., 1987; Pascual-Marqui et al., 1995). Using the cross validation criterion and subsequent topographical ANOVA, we determined that the optimal number of microstates to best describe the current data set was four.

For the K-means clustering algorithm used in the present study, the topographies at the GFP maxima were first concatenated across both the narcolepsy patients and controls. Previous studies have typically concatenated topographies for patients and controls separately (Wackermann et al., 1993; Brandeis et al., 1995; Lehmann et al., 1998; Cantero et al., 1999; Galderisi et al., 2001; Katayama et al., 2007; Lehmann and Michel, 2011), however, we chose to concatenate the topographies across the entire group, both to ensure that any between-group comparisons were valid and to facilitate correlation with the resting-state fMRI results. Second, four topographies were randomly selected from the concatenated data to form “template topographies”. Third, the template topographies were then spatially correlated with each time point in the concatenated data set, yielding a spatial correlation value. From these spatial correlation values, the GEV was calculated. The GEV quantifies how well each of the four templates describes the whole data set (Pascual-Marqui et al., 1995). Each of the four template topographies was then averaged with all topographies from those data points where it yielded the highest spatial correlation. Finally, the spatial correlation and GEV for these redefined template topographies were recalculated. This procedure of selecting new template topographies and calculating the spatial correlation and GEV was iteratively repeated until the GEV became stable (Murray et al., 2008). The K-means clustering analysis yielded four microstates topographically similar to those previously described in the literature (Wackermann et al., 1993; Pascual-Marqui et al., 1995), which were then fitted back competitively using spatial correlation to the original EEG data. The microstate analysis—as described above for the present study—was performed using “Cartool”^2^, a dedicated software package for automatic microstate-segmentation of EEG data (Murray et al., 2008).

As described in the “Introduction” Section, in addition to the microstate topographies, the microstate analysis also results in several temporal related parameters for each microstate, including mean duration, ratio of total time, and transition probability. To review, mean duration is a measure of the average number of consecutive time frames occupied by any given microstate (here expressed in milliseconds). This can also be described as the mean dwell time for each microstate. Transition probability is the measure of the likelihood of transitioning from the current microstate to any other microstate or to remain in the same microstate. The ratio of total time is a measure of each microstate topography’s temporal presence over the entire data set.

### Statistical Comparisons of EEG Microstate Parameters

To test our main study hypothesis that the narcolepsy patients would exhibit decreased mean duration of the microstates compared with the age and gender matched controls, a repeated-measures ANOVA was performed simultaneously comparing across all four microstates. Age at time of MRI scanning and gender were included as covariates to ameliorate any potential developmental and sex-related differences in brain function. Overall multivariate significance for mean duration was assessed at *p* < 0.05, using the Wilks’ Lambda correction. *Post hoc* comparisons on the unstandardized residuals were considered significant at *p* < 0.0125 (e.g., *p* < 0.05, with Bonferroni correction for comparing across the four microstates). As this is the first study to use EEG microstate analysis to examine resting-state brain function in adolescents with predominantly type 1 narcolepsy, we performed three additional exploratory repeated-measures ANOVAs for the remaining EEG microstate temporal related parameters: GEV, ratio of total time, and transition probability to investigate any informative differences that may exist for these parameters. Again, age at time of MRI scanning and gender were included as covariates. For these exploratory analyses, we considered multivariate significance at *p* < 0.1, using the Wilks’ Lambda correction, and *post hoc* significance of the unstandardized residuals at *p* < 0.0125. All statistical analyses were performed in SPSS v22 (release 2013) for Macintosh (IBM Corporation, Armonk, NY, USA).

### fMRI Analysis

All fMRI images were reconstructed on the scanner and realigned using the SPM toolbox, INRIAlign (Freire and Mangin, 2001; Freire et al., 2002), on a per-participant basis. Each participant’s translation and rotation correction parameters were individually examined to ensure that no participant had significant head motion larger than one voxel in any direction. No participants were excluded for head motion. Spatial normalization into Montreal Neurological Institute (MNI) space was initially performed on the mean functional image volume for each participant, and these normalization parameters were then applied to each respective functional image set. The normalized images were smoothed with an 8 mm full width half maximum (FWHM) Gaussian kernel. All preprocessing was performed using SPM8^3^.

Functional connectivity was calculated using a group ICA algorithm (Calhoun et al., 2001), as implemented in the GIFT 2.0a toolbox^4^. A single ICA was performed on all 32 participants, with back reconstruction of single-subject spatial maps and time-courses from the raw data (Erhardt et al., 2011). To reduce computational load, two primary component analysis data reduction steps were performed: first, at the individual subject level and second, at the group level. Thirty-eight spatial components with associated time courses were estimated using the Infomax algorithm (Bell and Sejnowski, 1995), where the number of components contained in the data was determined using the minimum length description (MDL) criteria adjusted to account for correlated samples (Li et al., 2007). Based on spatial correlation with gray matter, white matter, and CSF maps, 11 components were determined to be artifacts. Both the choice of preprocessing steps and group ICA analytic procedures were performed in accordance with the best practices laid out by Allen et al. (2011) for the GIFT group ICA toolbox.

To further reduce the search area, the remaining 27 components were compared to the so-called canonical set of RSNs consistently reported in the literature (e.g., Damoiseaux et al., 2006; Mantini et al., 2007; Laird et al., 2011), resulting in the retention of 15 components. To visualize the components, voxel-wise one-sample *t*-test statistical parametric maps (shown at *p* < 0.05, family wise error (FWE) corrected for comparing across all voxels in the brain) that represented the regional strength of functional connectivity across the entire sample were created (Figure 4, Table 4). Additionally, between-group, voxel-wise two-sample *t*-tests were conducted on the 15 independent components to determine whether any narcolepsy-related differences in resting state functional connectivity were present.

### fMRI—EEG Analysis

To determine the relationship between the fMRI resting state components and the EGG microstates, the time courses for each of the four EEG microstate topographies identified from the K-means clustering analysis for each subject were first down sampled to match the temporal sampling of the fMRI data. Then, the onsets and durations of each of the four topographies were extracted for each participant individually. These onsets and durations were used as input to the multiple regression temporal sorting algorithm in the GIFT toolbox. The temporal sorting algorithm in GIFT (Calhoun et al., 2009) first convolves the input timings with the canonical hemodynamic response function to create SPM-type regressors before calculating, on an individual subject level, the slopes of the regressors between the time courses of each of the four EEG microstates and the time courses of each of the 15 RSNs of interest. These slopes were then averaged for each RSN and EEG microstate pairing for the narcolepsy patients and the healthy controls separately, converted to standardized Z-scores, and displayed as a heat map. Only those pairings with a Z-score of at least one were considered for interpretation. Also, given that no previous study has discussed the implications of an EEG microstate being negatively correlated with an fMRI derived RSN, negative slope values are displayed but not discussed.

### Sleep Scoring

Sleep was scored according to modified AASM criteria (Iber and American Academy of Sleep Medicine, 2007), using 30 s epochs. As the EEG recording cap did not provide electrooculography (EOG) channels, fronto-polar electrodes were used to detect eye movements. As no surface electromyogram (EMG) electrodes were available, onset of REM sleep was defined as the first REM with an EEG that fulfilled AASM criteria (low-amplitude, mixed-frequency background activity). End of REM sleep was defined as the first occurrence of a sleep spindle, a K complex, an EEG arousal, or a transition to sleep stage three sleep (Iber and American-Academy-of-Sleep- Medicine, 2007).

## Results

### Demographic Data

As stated previously, the narcolepsy patient group and control group did not differ in terms of either age or gender. We did note that the narcolepsy patients had a higher overall body mass index (BMI) compared with the healthy controls (*p* < 0.039), where the narcolepsy patients had a mean BMI of 25.6 and the controls 21.7.

### Sleep-Behavior Data

Both patients and controls used the actigraphy device for a mean of 6 days during the week prior to their MRI scan. We found no difference between patients and controls in daily mean sleep duration, which was 6 h and 15 min for all participants. The patients, however, spent more time lying down (*p* < 0.001) the week before the examination and had lower sleep efficacy, 63% in narcolepsy and 82% in controls (*p* < 0.01). The patients also scored higher on ESS than the controls (*p* < 0.001). No between-group differences were noted in fatigue, depression, anxiety or sleepiness measured just prior to scanning.

### EEG Microstate Parameters

**Figure 1:**
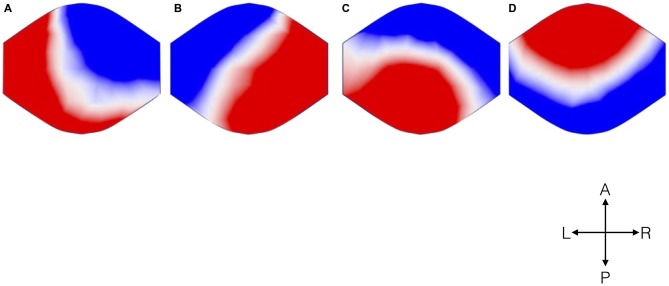
**Topographical microstate maps.** The figure shows the resulting four microstates, which are labeled **(A–D)** according to previous literature. The different colors signify the different polarities. The maps are represented as seen from above (A: Anterior, P: Posterior, L: Left, R: Right).

Analysis of EEG data resulted in four microstates topographically similar to those previously described in the literature (Figure 1). The different colors signify different polarities of the voltage over the scalp, represented as seen from above.

#### Mean Duration

**Figure 2:**
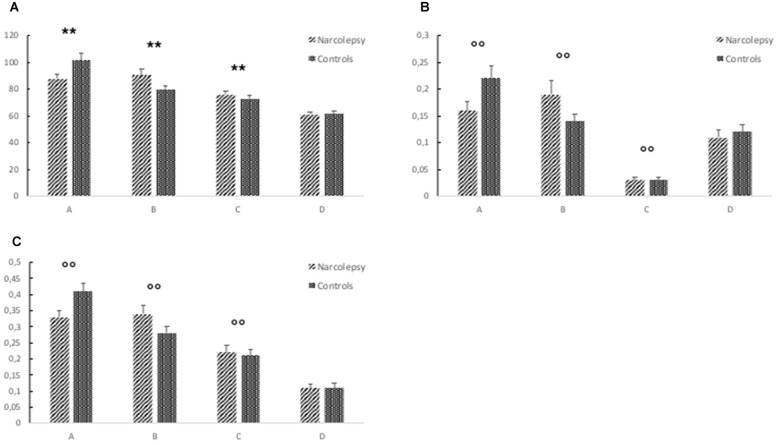
**Results from the electroencephalography (EEG) microstates analysis.** The figure shows **(A)** mean duration of each microstate (in ms), **(B)** mean global explained variance (GEV), and **(C)** ratio of total time covered for each microstate. The error bars represent standard error. Descriptive data can be found in Table 3. “**” Indicates a significant *post hoc* difference. “°°” Indicates a trend-level *post hoc* difference.

For mean duration of microstates, there was a significant overall multivariate omnibus effect for the interaction of group × microstate (*F*_(3,26)_ = 3.527, *p* < 0.029). No significant effects were noted for either age or gender. *Post hoc* tests indicated that the healthy controls spent more time in Microstate A than the narcolepsy patients (*t* = 6.11, *p* < 1.03 × 10^−6^). The narcolepsy patients were found to have significantly greater mean duration than the healthy controls for Microstate B (*t* = 6.91, *p* < 1.18 × 10^−7^) and Microstate C (*t* = 8.81, *p* < 7.96 × 10^−10^). No differences were noted for Microstate D (Figure 2A, Table 3).

**Table 3:** **Descriptive data of the EEG microstates analysis**.

Map/Parameter	Patients	Controls	*T*-statistic	*P*-value
Mean duration* (ms)
A	87.58	101.44	6.11	1.03E-06
B	90.47	80.02	6.91	1.13E-07
C	75.92	72.92	8.81	7.96E-10
D	61.14	61.54		n.s.
**Global explained variance^†^ (GEV)**
A	0.16	0.22	9.13	3.68E-10
B	0.19	0.14	5.09	1.81E-05
C	0.03	0.03	37.21	1.15E-26
D	0.11	0.12		n.s.
**Ratio of total time covered^†^**
A	0.33	0.41	7.70	1.36E-08
B	0.34	0.28	4.39	1.31E-04
C	0.22	0.21	17.12	4.94E-17
D	0.11	0.11		n.s.

The table shows the mean duration in milliseconds (ms), mean global explained variance, and ratio of total time for each microstate (A–D). *Indicates a significant multivariate effect (p < 0.05, corrected for comparing across one parameter). ^†^Indicates a multivariate statistical trend (p < 0.1, uncorrected).

**Table 4:** **Description of resting state networks (RSNs)**.

	**BA**	*x*	*y*	*z*	*T*
**Comp 1**
Left Pre-/Postcentral gyrus	6	−54	−10	30	25.89
Right Pre-/Postcentral gyrus	6	66	−2	24	20.79
**Comp 2**
R Posterior cingulate	29	8	−48	14	35.52
R Cingulate gyrus/Precuneus	31	4	−60	28	28.28
L Cingulate gyrus/Precuneus	31	−2	−54	30	33.36
R Cingulate gyrus	23	4	−22	28	28.77
**Comp 3**
R Calcarine	18	18	−104	0	31.73
L Calcarine	18	−18	−104	−4	30.72
R Hippocampus/Parahippocampus		14	−30	−4	6.73
L Posterior cingulate		−12	−56	14	7.24
**Comp 4**
L Cerebellum		−12	−72	−12	18.8
L Lingual gyrus	18	−14	−80	−14	19.76
R Lingual gyrus	18	18	−80	−10	18.48
R Cerebellum		26	−86	−20	17.62
**Comp 5**					
Anterior cingulate/Superior frontal gyrus	32, 10	−4	44	4	29.26
Precuneus	31	−2	−58	28	28.51
L Middle temporal/ Angular gyrus	39	−52	−66	28	12.78
R Middle temporal/ Angular gyrus	39	56	−60	28	10.78
L Orbital frontal gyrus	47	−42	20	−18	12.66
R Orbital frontal gyrus	47	40	12	−12	7.35
**Comp 6**
R Middle temporal gyrus		58	−62	0	27.44
L Middle occipital/Temporal gyrus		−46	−78	2	19.58
**Comp 7**
Cingulate gyrus	24, 32	2	2	44	13.00
Left superior temporal lobe	22	−46	2	−4	25.91
Right superior temporal lobe	22	56	−4	−2	22.36
**Comp 8**
R Parahippocampus/Hippocampus/ Amygdala		20	2	−22	35.49
L Parahippocampus/Hippocampus/ Amygdala		−20	4	−16	20.08
**Comp 9**
R Postcentral gyrus/ Supramarginal gyrus		56	−26	40	39.13
L Postcentral gyrus		−50	−30	44	22.42
R Inf frontal gyrus	44	54	8	16	10.80
L Inf frontal		−50	6	14	9.16
L Middle frontal		−38	42	18	11.22
R Middle frontal		36	40	16	7.65
R Inf temporal		52	−66	−6	9.02
L Inf temporal	37	−48	−66	−2	9.37
R Culmen		24	−54	−26	8.01
L Culmen		−26	−54	−28	11.12
**Comp 10**
L Putamen		−16	16	−4	38.64
R Caudate		12	20	−6	34.16
ACC	32	−4	36	−6	28.66
**Comp 11**
Supplementary motor area/ Middle cingulate gyrus	6, 24	0	−6	50	35.33
Left precentral gyrus	4, 6	−36	−16	46	19.97
Right precentral gyrus	6, 4	30	−16	48	21.28
**Comp 12**
ACC	24, 32	−4	24	28	26.92
L Middle frontal gyrus	9, 1	−30	33	30	20.07
R Middle/Superior frontal gyrus		32	44	28	14.90
L Insula		−40	12	−4	13.07
R Insula		56	12	−4	15.79
L Inferior frontal gyrus		−52	4	20	10.23
**Comp 13**
Medial occipital lobe/Lingual gyrus	18	−2	−94	−6	40.91
**Comp 14**
R Caudate/Putamen		16	6	8	23.38
L Caudate/Putamen		−10	4	4	27.12
L Putamen		−28	−18	0	19.25
R Putamen		28	−16	−2	20.16
ACC		−4	32	8	9.05
L Inf frontal		−48	26	6	7.23
L Thalamus		−8	−26	−6	15.91
R Thalamus		10	−14	−2	16.64
R Caudate		16	16	2	28.35
L Caudate		−10	8	4	25.99
**Comp 15**
L Middle/Superior temporal gyrus	39	−48	−60	16	19.32
L Precuneus		−22	−62	16	19.13
R Precuneus		16	−56	14	17.24
R Middle/Superior temporal	22	50	−62	14	17.49
Precuneus	7	−4	−54	46	16.06

*The table shows the peak Montreal Neurological Institute (MNI) stereotactic co-ordinates (x, y, z) for the 15 resting state network components submitted to temporal sorting based on the four microstate time courses. BA, Brodmann area. T, statistic value of the independent component resting state analysis*.

#### Global Explained Variance (GEV)

There was a trend level multivariate effect for the interaction of group × microstate (*F*_(3,26)_ = 2.43, *p* < 0.088) for the GEV of each microstate. No significant effects of either age or gender were noted. *Post hoc* tests suggested that for the healthy controls, Microstate A explained more of the GEV than for narcolepsy patients (*t* = 9.13, *p* < 3.68 × 10^−10^). For the narcolepsy patients, Microstates B (*t* = 5.09, *p* < 1.81 × 10^−5^) and C (*t* = 37.2, *p* < 1.15 × 10^−26^) were found contain more of the GEV than for healthy controls (Figure 2B, Table 3). No other differences were noted.

#### Ratio of Total Time

For the ratio of total time, there was also a trend-level multivariate effect for the interaction of group × microstate (*F*_(3,26)_ = 3.214, *p* < 0.039). Again no effects of age or gender were noted. *Post hoc* tests showed that the healthy controls had a larger RTT for Microstate A than the narcolepsy patients (*t* = 7.7, *p* < 1.37 × 10^−8^). For the narcolepsy patients, Microstates B (*t* = 4.39, *p* < 1.31 × 10^−4^) and C (*t* = 17.12, *p* < 4.9 × 10^−17^) were found to have greater RTT than for the controls (Figure 2C, Table 3). No differences were observed for Microstate D.

#### Transition Probability

**Figure 3:**
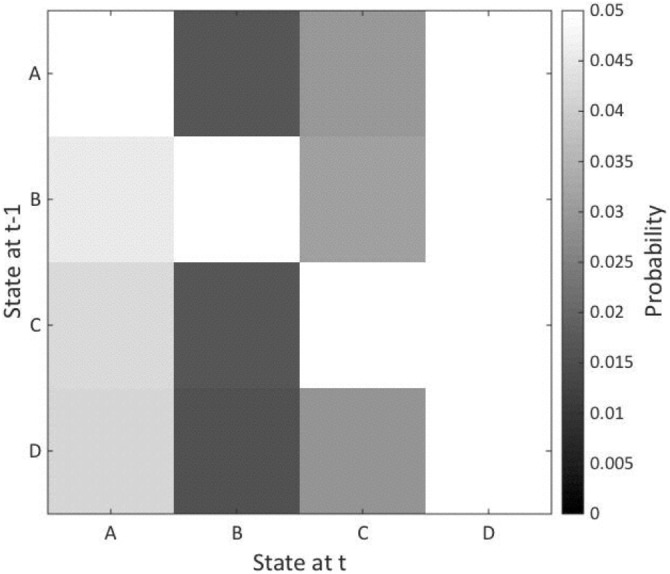
**Microstate transition probability.** The figure shows the probability of transition from one microstate to another with the results of the patients subtracted from the healthy controls. The vertical axis represents states at time point −1 and the horizontal axis shows states at time 1. The microstates are indicated A–D. No significant between-group differences were noted.

No significant multivariate effect for the interaction of group × microstate was noted for transition probability (*F*_(13,16)_ = 1.27, ns). Nor were any significant effects of age at time of MRI scan or gender observed (Figure 3).

### fMRI-EEG Correlation

The ICA analysis of the resting state fMRI data resulted in 27 components, 15 of which were retained for temporal correlation with the EEG microstates (Figure 5, Table 5). No significant between-group differences were observed at the whole brain level for any of these 15 independent components.

**Figure 4:**
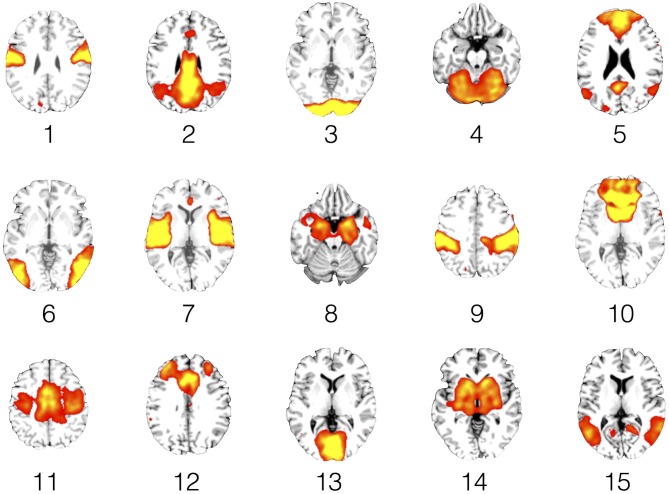
**Resting state networks (RSNs).** This figure shows representative slices for each of the 15 RSNs. The functional connectivity maps are rendered as 1-sample *t*-tests (*p* < 0.05, family wise error (FWE) corrected for comparing across the whole brain) across the entire study sample of narcolepsy patients and healthy controls.

**Figure 5:**
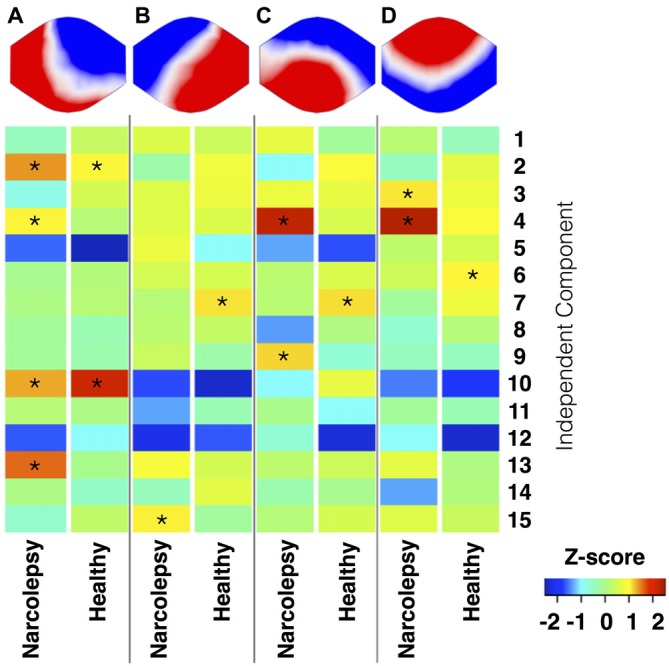
**Temporal correlation of EEG microstates and functional magnetic resonance imaging (fMRI) RSNs.** This figure shows the results of the temporal sorting of the RSNs using the time courses of the four EEG microstates. Results are stratified in terms of microstate and study group, with results for the narcolepsy patients and controls displayed separately. The temporal sorting regression coefficients are displayed in terms of Z-scores. *Indicates a temporal correlation with a Z-score >1.

**Table 5:** **Summary of the temporal correlation analyses between the EEG microstates and the fMRI RSNs**.

	Microstate A		Microstate B		Microstate C		Microstate D	
Component #	Narcolepsy	Healthy	Narcolepsy	Healthy	Narcolepsy	Healthy	Narcolepsy	Healthy
1	−0.47	0.35	0.62	0.41	0.76	−0.16	0.18	−0.46
2	1.41	1.04	−0.32	0.86	−0.95	0.97	−0.49	0.70
3	−0.81	0.54	0.64	0.82	0.81	0.76	1.09	0.81
4	1.04	0.14	0.67	0.61	2.25	0.58	2.37	0.97
5	−1.60	−2.51	0.80	−0.95	−1.35	−1.70	0.26	0.56
6	−0.09	0.11	0.52	0.55	0.21	0.61	0.47	1.04
7	0.00	0.16	0.16	1.11	0.19	1.13	−0.18	0.84
8	−0.14	−0.38	0.21	0.34	−1.39	0.04	−0.63	0.13
9	−0.15	−0.32	0.37	−0.31	1.16	−0.66	−0.47	−0.50
10	1.33	2.15	−1.72	−2.39	−1.01	0.76	−1.51	−1.86
11	0.14	−1.64E-04	−1.35	−0.40	−0.05	−0.95	−0.19	−0.40
12	−1.67	−0.95	−2.14	−1.66	−0.65	−2.27	−0.94	−2.36
13	1.59	−0.06	0.92	0.50	0.24	0.44	0.74	0.04
14	−3.44E-03	−0.53	−0.45	0.70	−0.33	−0.08	−1.35	0.07
15	−0.59	0.28	1.06	−0.20	0.13	0.54	0.65	0.41

*The table shows the z-score value of the temporal correlation between the time courses of each EEG microstate and the time courses of each of the 15 RSNs*.

#### Microstate A

We observed for healthy controls that Microstate A was temporally correlated with both the anterior (component 10) and posterior (component 2) aspects of the default mode network. For the narcolepsy patients, in addition to the anterior (component 10) and posterior (component 2) aspects of the default mode network, Microstate A was also temporally correlated with a component comprising the anterior cerebellum (component 4) and one encompassing the primary visual cortex (component 13) component. Results are summarized in Figure 5.

#### Microstate B

Microstate B was found to be temporally correlated (Figure 5) with the primary auditory cortices (component 7) in the healthy controls. For the narcolepsy patients, Microstate B was found to be correlated with an independent component composed of precuneus and middle/superior temporal gyri (component 15).

#### Microstate C

For healthy controls, microstate C was observed to be temporally correlated (Figure 5) with the primary auditory cortices (component 7). In contrast, Microstate C was observed to be temporally correlated with independent components comprising the anterior cerebellum (component 4) and the postcentral, middle, and inferior frontal gyri (component 9).

#### Microstate D

Microstate D was found to be temporally correlated (Figure 5) with bilateral middle temporal gyri (component 6) in the healthy controls. For the narcolepsy patients, Microstate D was found to be correlated with both the secondary visual cortices (component 3) and the anterior cerebellum (component 4).

### Sleep During Examination

**Figure 6:**
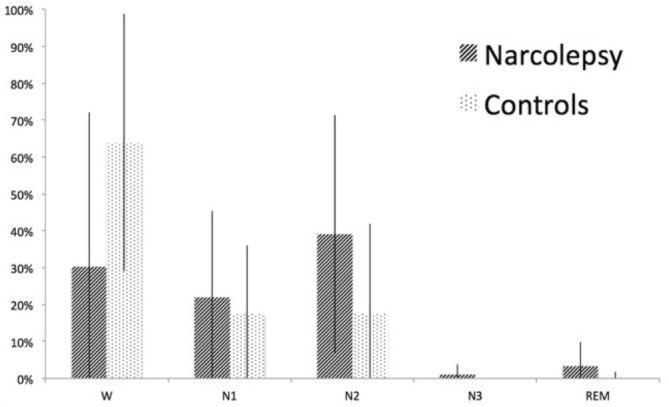
**Sleep stages during the fMRI-EEG examination.** The figure shows percent time spent in wake (W), sleep stage 1 (N1), sleep stage 2 (N2), sleep stage 3 (N3), and rapid eye movement (REM) sleep for narcolepsy patients and controls, respectively. The error bars represent the standard deviation.

Both patients and controls fell asleep during the examination. The controls spent twice as much time awake as the patients, while the patients spent more of their sleep in sleep stage 2 (Figure 6). However, neither of these differences was statistically significant (*p* > 0.1). Duration and occurrence of microstates were not found to correlate with either sleep or wakefulness during the examination. Similarly, neither sleep nor wakefulness was found to correlate with any of the time courses of the 15 RSNs of interest.

## Discussion

The aim of this study was to investigate and describe resting state brain function in adolescents with predominantly type 1 narcolepsy using simultaneous fMRI-EEG. Four microstates were identified resembling those previously described in the literature and the main findings of this article can be summarized as follows: (1) The mean duration of Microstate A was shorter in patients, while the mean duration of Microstates B and C was longer in patients. (2) Similarly, there was also a trend for patients to spend less of the total scan time in Microstate A, where Microstate A explained less of the variance in the data of the narcolepsy patients. Patients were more likely to spend more of the total scan time in Microstates B and C, where these microstates also explained more of the variance of the data in the patients. (3) Narcolepsy patients and healthy controls had similar but not identical mappings between individual microstates and fMRI derived RSNs. Specifically of interest, the time course of Microstate A correlated most strongly with the time courses of components representing the anterior and posterior aspects of the default mode network in healthy controls. In narcolepsy patients, though, Microstate A was found to correlate not only with the same default mode components but also with cerebellar and primary visual cortex components.

### Microstates

Recalling that mean duration is thought to reflect the stability of a microstate’s underlying neural generator (Dierks et al., 1997; Stevens et al., 1997), in this study, we found that patients had significantly lower mean duration for Microstate A and significantly higher mean duration for Microstates B and C compared with the controls. In support of this result, we also noted trends for Microstate A to cover less of the total time, as well as explaining less of the variance in the data, in narcolepsy patients compared with the healthy controls. Whereas Microstates B and C were observed to cover more time and explain more of the variance of the data for the patients. Despite these latter differences (GEV, RTT, and transition probability) not showing a significant overall multivariate effect, there seemed to be a consistent pattern for narcolepsy patients to have underlying instability of Microstate A and more stability of Microstates B and C. Our results lie in contrast to those reported previously in a study of adults with narcolepsy (Kuhn et al., 2015), where the authors found that Microstate D was found to play an important role in sleep and wake in narcolepsy, while controls were found to rely more on Microstate C. Additionally, our general pattern of findings for both significant and trend-level between-group differences for mean duration, GEV, and ratio total time for Microstates A, B, and C, appears not to parallel with those of Kuhn et al. (2015), who reported that mean duration, GEV, ratio total time, and transition probability were largely similar in both controls and narcolepsy patients during wakefulness and sleep. They did find evidence for reduced mean duration of Microstates B and C in the narcolepsy patients during sleep, which they interpreted as evidence of instability of sleep regulation in narcolepsy (Kuhn et al., 2015). The authors noted no differences in mean duration during wakefulness for any of the microstates. Given that Kuhn et al. (2015) focused primarily on EEG microstate dynamics during sleep in adults with narcolepsy and our study was concerned more with EEG microstate dynamics during unstructured rest in adolescents with narcolepsy, it is perhaps unsurprising that our results are so disparate from those previously reported (Kuhn et al., 2015).

### Microstates and Sleep

A general concern when investigating resting state is that the participants may fall asleep during the examination (Tagliazucchi and Laufs, 2014). As microstate segmentation is dependent on activity mainly in the alpha band and alpha wave frequency is reduced in sleep (Cantero et al., 1999; Brodbeck et al., 2012), this could result in less GFP peaks during periods of sleep. Consequently, a reduced number of GFP peaks may lead to an increased duration of microstate maps as has been shown by other investigators (Wehrle et al., 2007; Brodbeck et al., 2012). Our approach to control for sleep during examination was to include sleep scoring in the analysis to determine if there were any significant correlations between microstates and the different sleep stages. We also compared the sleep-scoring results between patients and controls for differences that may explain our results (Figure 6). As can be seen in Figure 6, sleep was over-represented in the narcolepsy group, but we note that the controls also slept during the examination. While there seemed to be a difference in sleep depth between controls and patients, this difference was not statistically significant. To attempt to further rule out sleep dependent differences, we extracted periods of wake and sleep stage 1 of at least 2.5 min in all participants where this could be found and re-ran the microstate analysis on the extracted epochs. The decision to include sleep stage 1 was based on the reports of two previous articles showing no difference in any of the microstate parameters between awake and sleep stage 1 (Wehrle et al., 2007; Brodbeck et al., 2012; Kuhn et al., 2015). This resulted in eight patients that were sleep-matched with eight healthy controls. Our results indicated a similar pattern of differences regarding Microstate A between the two groups, but the small sample size prevented us from detecting any significant differences. While we understand the necessity to exercising caution in the interpretation of these results, we feel they are important to include as it may give an indication of a difference that may be narcolepsy specific, and we therefore suggest that future studies aim to investigate longer periods of wakefulness during unstructured rest in narcolepsy patients.

### Microstates and fMRI Resting State Networks

The results from the temporal sorting of the fMRI derived RSNs based on the time courses of the four EEG microstates indicated that the narcolepsy patients and healthy controls had similar but not identical relationships between individual microstates and RSNs. In healthy controls, Microstate A correlated most strongly with components comprised of both the anterior and posterior aspects of the default mode network (Laird et al., 2011). In narcolepsy patients, Microstate A correlated not only with the same default mode components but also with a primary visual cortex component. Given the current lack of consensus in the field in terms of the relationship between EEG microstates and fMRI derived RSNs (Britz et al., 2010; Van de Ville et al., 2010; Yuan et al., 2012), a certain level of care must be taken in interpreting the findings presented here. That being said, our results appear to suggest that narcolepsy patients engage both the anterior and posterior aspects of the default mode network less than healthy controls. The results also suggest that multiple neural networks may be contributing to the electrical field configuration for Microstate A in the narcolepsy patients, which may indicate abnormal resting state brain dynamics. Given that we observed a similar pattern of between-group differences for Microstate A when looking only at the time spent in wake and sleep stage 1 and found no significant difference in sleep between the two groups, our findings indicate that this might be an underlying functional disturbance specific to narcolepsy. Activity within the default mode network has been shown to relate to numerous cognitive, autobiographical memory, emotional, and self-referential functions (e.g., Buckner et al., 2008; Spreng et al., 2009; Laird et al., 2011; Menon, 2011), making it tempting to overreach in our interpretation of this finding as underpinning all self-report cognitive complaints and measured cognitive deficits, such as sustained attention, in narcolepsy patients (Rieger et al., 2003; Naumann et al., 2006; Bayard et al., 2012). Before such a claim is made, though, future studies are needed in larger populations of narcolepsy patients to both verify this EEG microstate-RSN relationship, as well as its relation to clinical symptomatology.

The remaining three microstates exhibited much weaker and much less specific temporal correlations with the 15 RSNs. However, in general, we observed a more conventional one-to-one mapping for the healthy controls, while the narcolepsy patients tended to have several RSNs mapping to each of the remaining microstates. Microstate B, which appeared to be more stable in the narcolepsy patients, correlated most strongly for narcolepsy patients with a RSN comprised of the precuneus and middle and superior temporal lobes. This particular RSN has been previously linked to higher order visual processing, such as viewing complex or emotional stimuli (e.g., faces and films), visual tracking of moving objects, and mental rotation (Laird et al., 2011). We also note that for the narcolepsy patients, Microstates C and D both strongly correlated with the RSN corresponding to bilateral anterior cerebellum, suggesting that these two microstates may not be entirely independent of each other. Similarly, Microstates B and C were both found to be strongly correlated with the RSN corresponding to the primary auditory cortices in controls, again suggesting that these two microstates may not be completely independent of each other. This may add further evidence to an apparent dysfunction in resting state brain activity dynamics in narcolepsy, as our attempt to link the various EEG microstates with fMRI derived RSNs seemed to indicate that for the narcolepsy patients, the four EEG microstates had different underlying neural generators than for healthy controls.

### Strengths and Limitations

One limitation of the study was the small sample size and the associated low statistical power. Narcolepsy is a disease with a low incidence in Sweden (Szakács et al., 2013) making recruitment necessarily limited, this, especially, as our study aimed at investigating adolescents only. The limited sample size meant that we did not have enough statistical power to examine only periods of wakefulness in all subjects. While this does raise questions about whether we would have observed stronger or different between-group changes had we not collapsed our analyses across both wakefulness and sleep, we point out that when we re-ran our microstate analysis on continuous periods of wakefulness and/or sleep stage 1 where it could be found (eight patients and eight controls), we observed the same pattern of results. We also note that while two studies have observed differences when comparing the microstate topographies across sleep stages (Brodbeck et al., 2012; Kuhn et al., 2015), spatial correlation analyses run by both studies indicated that the EEG microstate topographies were at least 70% similar across both wakefulness and sleep stages 1 and 2 and that the biggest differences in topography were noted for Microstates C and D. Given that microstate topography is thought to be a proxy for the underlying neural generator and Microstates A and B shared at least 90% similarity across wakefulness and sleep stages 1 and 2, we feel confident that our decision to collapse our microstate analysis across both wakefulness and sleep should not have had a significant effect on our results regarding Microstates A and B. Additionally due to ethical restrictions, we were unable to ask participants to refrain from taking any medication prior to participating in the study. This may be a concern as some medications have been demonstrated to improve cognitive function (Schwartz et al., 2004; Saletu et al., 2009; Esposito et al., 2013; Cera et al., 2014), which may further explain why we were unable to detect more significant differences in the microstate analysis.

## Conclusion

In this study we attempted to investigate and describe resting state brain function in adolescents with predominantly type 1 narcolepsy using simultaneous fMRI-EEG. We found that narcolepsy patients had significantly lower mean duration than controls for Microstate A, a microstate which we found to be related to both the anterior and posterior aspects of the default mode network and may suggest a disruption of this network that is disease specific.

## Author Contributions

NMD: all data acquisition, analysis of EEG data, interpretation of data, principal writer of the manuscript; AS: design of the work, patient recruitment, drafting the work; STW: analysis of fMRI data, interpretation of data, drafting and revising the work; AW: data acquisition, analysis of behavioral data, drafting the work; MU: analysis of EEG data, drafting the work; HS: analysis of MRI data, drafting the work; ND: conception of the work and revising it critically; TH: patient recruitment, conception and design of the work and revising it critically; A-ML: patient recruitment, conception and design of the work and revising it critically; ME: conception and design of the work, data acquisition and analysis, interpretation of data, drafting the work and revising critically for important content. All authors have made final approval of the version to be published and agree to be accountable for all aspects of the work in ensuring that questions related to the accuracy or integrity of any part of the work are appropriately investigated and resolved.

## Funding

The Research Council of South East Sweden (FORSS), the Knut and Alice Wallenberg foundation (KAW), the strategic research area of systems neurobiology at Linköping University, and the Country council of Östergötland Sweden are acknowledged for financial support of the study.

## Conflict of Interest Statement

The authors declare that the research was conducted in the absence of any commercial or financial relationships that could be construed as a potential conflict of interest.

## Footnotes

^1^http://www.stanfordhealthcare.org

^2^http://brainmapping.unige.ch/cartool

^3^http://www.fil.ion.ucl.ac.uk/spm

^4^http://icatb.sourceforge.net

## Abbreviations

BOLD: blood oxygen level dependent
ECG: electrocardiogram
EEG: electroencephalography
ESS: Epworth sleepiness scale
fMRI: functional magnetic resonance imaging
GEV: global explained variance
GFP: global field power
PET: positron emission tomography
REM: rapid eye movement
RSN: resting state network
SPECT: single-photon emission computed tomography
TTL: Transistor-Transistor Logic
VAS: visual analog scale.
