# Corrigendum: Altered Brain Microstate Dynamics in Adolescents With Narcolepsy

**DOI:** 10.3389/fnhum.2019.00385

**Published:** 2019-11-06

**Authors:** Natasha M. Drissi, Attila Szakács, Suzanne T. Witt, Anna Wretman, Martin Ulander, Henriettae Ståhlbrandt, Niklas Darin, Tove Hallböök, Anne-Marie Landtblom, Maria Engström

**Affiliations:** ^1^Department of Medical and Health Sciences (IMH), Linköping University, Linköping, Sweden; ^2^Center for Medical Image Science and Visualization (CMIV), Linköping University, Linköping, Sweden; ^3^Department of Paediatrics, Institute of Clinical Sciences, Sahlgrenska Academy, University of Gothenburg, Gothenburg, Sweden; ^4^Department of Behavioral Science and Learning, Linköping University, Linköping, Sweden; ^5^Department of Clinical and Experimental Medicine, Linköping University, Linköping, Sweden; ^6^Department of Radiology, Medical Diagnostics, Highland Hospital, Eksjö, Sweden; ^7^Department of Neurology, Uppsala University, Uppsala, Sweden

**Keywords:** narcolepsy, default mode network, functional magnetic resonance imaging (fMRI), electroencephalography (EEG), microstates, resting state networks, orexin, sleep

In the original article, there was a mistake in the legend for Figure 2 as published. The legend states that the error bars represent the standard deviation, this is incorrect. The error bars in Figure 2 represent the standard error. The correct legend appears below.

**Figure 2:**
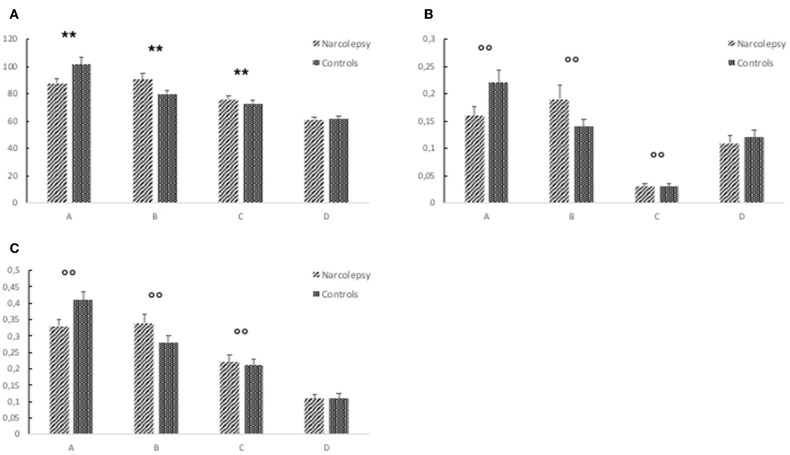
Results from the electroencephalography (EEG) microstates analysis. The figure shows **(A)** mean duration of each microstate (in ms), **(B)** mean global explained variance (GEV), and **(C)** ratio of total time covered for each microstate. The error bars represent the standard error. Descriptive data can be found in Table 3. “^**^” Indicates a significant *post hoc* difference. “^°°^” Indicates a trend-level *post hoc* difference.”

Additionally, in the original article, there was a mistake in Figure 2 as published. The figure legend indicating which bars represent narcolepsy and control have been reversed, so that the diagonal stripes are incorrectly shown to represent the narcolepsy group while the dots represent the control group. This should be reversed to be in line with the data in Table 3 as well as in the Results, where the group differences are described correctly. The corrected Figure 2 appears below.

The authors apologize for these errors and state that they do not change the scientific conclusions of the article in any way. The original article has been updated.

